# Differences in perceived intra-oral dryness in various dry-mouth patients as determined using the Regional Oral Dryness Inventory

**DOI:** 10.1007/s00784-020-03734-2

**Published:** 2021-01-26

**Authors:** Z. Assy, C. P. Bots, H. Z. Arisoy, S. S. Gülveren, F. J. Bikker, H. S. Brand

**Affiliations:** 1 Department of Oral Biochemistry, Academic Centre for Dentistry Amsterdam, University of Amsterdam and VU University Amsterdam, Room 12N-37, Gustav Mahlerlaan 3004, 1081 LA Amsterdam, The Netherlands; 2 Saliva Clinic of the Dutch Institute for Salivary Research, Bunschoten, Netherlands

**Keywords:** Dry mouth, Xerostomia, Salivary flow rate, Salivary pH, Xerostomia Inventory

## Abstract

**Objectives:**

Recently, it was shown that the Regional Oral Dryness Inventory (RODI) could determine differences in dry-mouth perception at different intra-oral locations. The main aim of this study was to determine whether the RODI might help to discriminate between various causes of oral dryness in dry-mouth patients. The second aim was to ascertain whether the RODI could become an additional diagnostic tool in dry-mouth patients.

**Materials and methods:**

Data were collected retrospectively from patients who visited a specialized saliva clinic. Salivary flow rates, Xerostomia Inventory scores, and RODI scores were extracted from the medical records. Patients were stratified into subgroups according to their health status.

**Results:**

Five hundred twenty-eight patients participated in this study (mean age of 59.6 ± 16.0 years; 68.4% female). Specific patient groups differed with regard to the region of the mouth they experienced as the most and least dry. The posterior palate was the area perceived as most dry by controls and Sjögren patients. In patients using limited or multiple medications, it was the anterior tongue. RODI scores also differed significantly among dry-mouth patient groups: whereas controls and patients using limited medication had the lowest RODI scores and experienced less intra-oral dryness, Sjögren patients had the highest RODI scores.

**Conclusion:**

Our use of the RODI questionnaire showed that perceived intra-oral dryness differed between the various dry-mouth patients.

**Clinical relevance:**

The RODI can be a valuable clinical diagnostic tool in dry-mouth diagnostics, in which it can be used to discriminate between the various causes of oral dryness in patients.

## Introduction

Saliva plays a crucial role in the preservation and maintenance of oral health due to its multiple functions, which include buffering capacity, lubrication, moistening, microbial homeostasis, and wound healing [1–4]. The consequences when salivary flow is impaired are therefore multidimensional, transcending oral health. For example, hyposalivation increases the risk of dental caries, gingivitis, and periodontitis. In addition, patients with impaired salivary flow can experience dry mouth, oral discomfort and pain, difficulty in speaking, taste alterations, and difficulty in swallowing [1, 2, 5]. Altogether, the effects of hyposalivation can have physical, emotional, and social impacts, thereby negatively affecting the quality of life, and particularly oral health [5, 6].

Dry-mouth symptoms can be caused by the use of xerogenic medications or multiple medications, but also by radiotherapy of the head and neck region, systemic diseases such as Sjögren’s syndrome, and chronic stress [1, 5, 7, 8]. Obviously, dry-mouth symptoms may also be induced by a combination of factors [5]. For example, multiple medication usage is common in patients with Sjögren’s syndrome. These etiologic factors produce dry-mouth symptoms through a variety of mechanisms. For example, dry-mouth–inducing medications have anticholinergic or sympathomimetic actions that affect the neural control of salivary glands, have a cytotoxic effect on the salivary glands, have a diuretic effect that depletes fluids, or damage the ion-transport pathways in the acinar cells. Irradiation of tumor sites in the head and neck region can also damage the salivary glands, leading to complete dysfunction of acini. On the other hand, Sjögren’s syndrome induces progressive immune-mediated self-destruction of the salivary glands and lacrimal glands [1, 5]. Several mechanisms thus lead to impaired salivary function, and, as a consequence, hyposalivation and xerostomia, i.e., perceived oral dryness.

As hyposalivation and xerostomia are not correlated per se [9, 10], any diagnosis of dry mouth should include objective parameters such as total salivary flow and subjective parameters such as total perceived oral dryness. However, due to the complex etiology of dry mouth and the various mechanisms underlying them, these parameters do not seem entirely discriminative. Diagnosis is difficult, especially for early-stage Sjögren’s patients who lack specific clinical manifestations and biomarkers [11]. As the median delay between the onset of Sjögren’s syndrome and diagnosis is 4 years (range 0–28 years) [12], these current diagnostic tools are not sufficient for a more advanced dry-mouth diagnosis.

Recently, it was shown that a new questionnaire, the Regional Oral Dryness Inventory (RODI), could be used to determine differences in dry-mouth perception at different locations in the mouth [13]. The study in question concluded that the dry-mouth feeling differed significantly among intra-oral locations, with the highest perceived oral dryness in the posterior palate and the lowest in the floor of the mouth. It was speculated that, clinically, the RODI might help to discriminate between different potential causes of oral dryness in patients. It was thus hypothesized that patients with oral dryness caused by irradiation of the head and/or neck region might have a different distribution of intra-oral dryness than those with Sjögren’s disease or medication-induced dry mouth [13].

The main aim of this study was therefore to determine whether the RODI might help to discriminate between causes of oral dryness in dry-mouth patients. To contribute to the study of dry-mouth diagnostics, the second aim was to ascertain whether the RODI might become an additional diagnostic tool in dry-mouth patients.

## Materials and methods

### Study design

Data for this retrospective case report study were collected from patients at the saliva clinic of the Dutch Institute for Salivary Research in Bunschoten, the Netherlands. They had been referred to this clinic by their dentists, general physicians, and medical specialists between October 2012 and April 2019. All patients had hyposalivation, xerostomia, hypersalivation, or other saliva-related problems. The study was approved by the Ethics Review Committee at the Academic Centre for Dentistry Amsterdam (ACTA, protocol number 201951). The reporting of this study conforms to the STrengthening the Reporting of OBservational studies in Epidemiology (STROBE) statement [14]. All data, questionnaires, and clinical variables were collected and interpreted by a single practitioner (CB) according to the standard operating procedures of the regular patient-care routine, which generally took approximately 25 min.

### Data collection methods

The relevant data were extracted from the medical record by two abstractors (HZA and SSG). The following clinical data were retrieved: age, sex, health status, number of medications used, Xerostomia Inventory (XI) scores, Regional Oral Dryness Inventory scores, salivary flow rate and salivary pH of unstimulated whole saliva (UWS), chewing-stimulated whole saliva (CH-SWS), and citric acid-stimulated whole saliva (A-SWS). The extracted data were pseudonymized so they could no longer be traced back to the patients.

Because some questionnaires or salivary variables were incomplete, the total number (*N*) for some of the collected data differs. After data entry, one researcher (ZA) verified that data transfer for all records was correct.

### Study variables

#### Questionnaires

When they visited the saliva clinic, all patients returned the prefilled questionnaires, including the Xerostomia Inventory (XI), the Regional Oral Dryness Inventory (RODI), and the European Medical Risk-Related History questionnaire. The XI consists of 11 items on a 5-point Likert scale ranging from 1 = “Never” to 5 = “Very often.” The items concern patients’ oral dryness and mouthfeel. Per item, patients indicate how often they experience problems regarding mouthfeel and oral dryness. The scores of the 11 items are summed to produce a total XI score that ranges between 11 (no xerostomia) and 55 (extreme xerostomia) [15].

The RODI questionnaire contains nine schematic illustrations of different locations in the oral cavity [13]. In our study, we used a slightly modified version with eight regions, excluding the throat. Four illustrations show areas in the upper jaw: the upper lip, the posterior part of the palate (from the rugae up to the end of the soft palate), the anterior part of the palate (including the rugae), and the inside part of the cheeks. The other four illustrations represent areas in the lower jaw: the lower lip, the anterior part of the tongue (from the tip of the tongue up to the vallate papilla), the posterior part of the tongue (from the vallate papilla up to end of the tongue), and the floor of the mouth. At each location, the patient uses a 5-point Likert scale ranging from 1 = “No dryness” to 5 = “severe dryness” to indicate the severity of the oral dryness they perceive [13].

The European Medical Risk-Related History questionnaire is an internationally validated patient-administered questionnaire that is used to map a patient’s current health status [16, 17]. On the basis of their health status, patients were allocated to the following groups: controls, patients using limited medication, patients using multiple medications, irradiated patients, irradiated patients using multiple medications, Sjögren syndrome patients, and Sjögren patients using multiple medications (see Table 1 for further details).

**Table 1:** Dry-mouth patient groups in this study listed on the basis of their health status. The abbreviation used per patient group is listed, together with the group’s health status

Patient groups	Abbreviation used in this study	Health status
Controls	Controls	None of the conditions listed below (i.e., radiation head and/or neck and Sjögren syndrome). Used no prescription medication
Patients using limited medication	Low Med patients	None of the conditions listed below. Used < 4 different prescription medications
Patients using multiple medications	High Med patients	None of the conditions listed below. Used ≥ 4 different prescription medications
Irradiated patients	RTX patients	Radiation of the head and/or neck area. Used < 4 different prescription medications
Irradiated patients using multiple medications	RTX + High Med patients	Radiation of the head and/or neck area. Used ≥ 4 different prescription medications
Sjögren syndrome patients	SS patients	Sjögren syndrome. Used < 4 different prescription medications
Sjögren patients using multiple medications	SS + High Med patients	Sjögren syndrome. Use ≥ 4 different prescription medications

Only prescription medications that were used on a structural basis were scored. We scored different types of medication, but not their dosages.

We did not score the following types of medication and self-medication: oily crèmes, Vaseline-like ointments, over-the-counter drugs, vitamins (even if they had been prescribed by a physician), nutritional supplements, homeopathic remedies, and medications or products to relieve dry mouth or dry eye (such as artificial tears or dry eye gel/ointment, pilocarpine tablets or eye drops, artificial saliva, and mouth-moistening gels or sprays). On the other hand, the following products were viewed as medication: corticosteroids or other anti-inflammatory crème/ointments and eye drops or eye gels with corticosteroid or other anti-inflammatory medicaments. But if a patient indicated clearly that he or she used over-the-counter anti-inflammatory drugs such as paracetamol or ibuprofen daily, these, too, were considered as medication.

### Sialometry and salivary pH

The patients were instructed not to eat, drink, chew gum, brush teeth, use mouthwash, or smoke at least 1 h before their visit to the saliva clinic. The salivary flow rate was determined as described in the following references [18, 19]. At the time of saliva collection, patients were placed in a quiet room and asked to sit in an upright position. The UWS was collected by the draining method in a pre-weighed plastic container [19]. Patients were asked to collect unstimulated saliva immediately after an initial swallow, by expectorating into the container as soon as they had collected the saliva in their mouth. During saliva collection, patients were not allowed to swallow. To collect CH-SWS, they were asked to chew a 5 × 5-cm sheet of parafilm (Parafilm M, Pechiney, Chicago, IL, USA) at a frequency of approximately 60 chews per minute and to expectorate into a pre-weighed plastic container every 30 s. To stimulate A-SWS secretion, a citric acid solution (2% *w*/*v*) was applied with cotton buds to the lateral borders of the tongue at 30-s intervals [20]. When the collection period had finished, the plastic containers were reweighted, and the collected volume was determined by subtracting the weight of the container before collection. Salivary flow was calculated by dividing the volume collected (assuming 1 g of saliva equals 1 mL) by the collection time (min). Salivary flow rates were expressed in mL/min [19]. To limit circadian variations, all patients were randomly assigned a time slot between 8:00 and 12:00 A.M. [21].

To determine whether patients suffered from hyposalivation, the following cut-off values were used: UWS < 0.10 mL/min, CH-SWS < 0.70 mL/min, and A-SWS < 0.70 mL/min [1].

The pH of saliva was measured and carried out immediately after saliva collection, within 5 min to minimize loss of CO_2_ to the atmosphere. The saliva pH was measured with pH paper (Merck KGaA, Darmstadt, Germany).

### Data analysis

The data were processed in Microsoft Excel and then converted into SPSS, version 25.0 (IBM Corp SPSS statistics, Armonk, NY, USA) for the statistical analysis. The Shapiro–Wilk test was used to assess the normality of the data. As not all variables were normally distributed, the data were presented as medians and their interquartile range (IQR). To clarify relatively small differences, the mean and standard deviation (SD) were also reported.

A Friedman test was conducted for the RODI scores of the total study population, followed by a Wilcoxon signed-rank test as a post hoc procedure.

The Kruskal-Wallis test was used to compare the different patient characteristics and RODI scores for all the various patient groups, followed by the Mann-Whitney *U* test as a post hoc procedure.

The possible relationship between the RODI scores and the total XI-scores was analyzed with a bootstrapped Spearman’s rank correlation test (1000 × bootstrapping). The Spearman’s rho coefficient and Bias-corrected accelerated (Bca) 95% confidence interval were extracted. The effect size of the correlation coefficient was interpreted as a negligible (*r* = 0.1–0.2), fair (*r* = 0.3–0.5), moderate (*r* = 0.6–0.7), or very strong (*r* = 0.8–0.9) correlation [22]. All significance levels (α) were set at 0.05.

## Results

### Total study population

A total of 528 health records were available. The mean age of participants in this study was 59.6 ± 16.0 years (*N* = 522; the age of 6 participants was not documented). Majority of the patients were female (68.4%) (*N* = 525; the gender of 3 participants was not documented). The RODI scores, XI-scores, UWS, CH-SWS, A-SWS salivary flow rates, and salivary pH were not normally distributed (Shapiro–Wilk test; *p* < 0.01). Table 2 presents the various total XI-scores, salivary flow rates, and salivary pH of the study population. The flow rates suggested that the following proportions of the study population were considered to have hyposalivation: UWS (33.4%), CH-SWS (55.2%), and A-SWS (29.6%). The mean number of medications used was 3 ± 4, with a median of 2 and IQR of 0–4 (*N* = 518; the number of medications was not listed for 10 participants).

**Table 2:** Patient characteristics for the total study population

Saliva		Mean ± SD	Median ± IQR	Number of subjects
UWS	Flow rate (mL/min)	0.21 ± 0.21	0.16 ± 0.07–0.30	434
	pH	6.38 ± 0.56	6.50 ± 6.10–7.00	416
CH-SWS	Flow rate (mL/min)	0.76 ± 0.62	0.60 ± 0.30–1.10	446
	pH	6.75 ± 0.58	7.00 ± 6.50–7.00	444
A-SWS	Flow rate (mL/min)	1.28 ± 0.92	1.11 ± 0.57–1.80	450
	pH	4.91 ± 1.04	4.60 ± 4.00–5.50	450
XI total score		31.8 ± 11.4	32.0 ± 23.0–40.0	507

The total *N* differs because some data were missing for some patients

The total XI-scores, the unstimulated whole saliva (UWS), chewing-stimulated whole saliva (CH-SWS), acid-stimulated whole saliva (A-SWS) flow rate (mL/min), and salivary pH of the study population. Data are expressed as the median with the corresponding interquartile range (IQR) and as a mean with standard deviation (SD)

### Regional Oral Dryness Inventory for the total study population

Table 3 shows the medians, corresponding IQRs, and means with standard deviations for each of the eight intra-oral regions of the RODI. Perceived oral dryness in the total study population differed significantly among the eight intra-oral regions (Friedman test *p* < 0.05, followed by Wilcoxon signed-rank test). The highest scores were found for the posterior part of the palate, and the lowest for the inside cheeks (Table 3).

**Table 3:** Perceived oral dryness in eight different intra-oral regions as determined with the Regional Oral Dryness Inventory (RODI) in the total study population

	Mean ± SD	Median ± IQR	Total number of subjects for each intra-oral region
Upper lip	2.84 ± 1.28	3.00 ± 2.00–4.00	449
Posterior part of palate^a^	3.04 ± 1.30	3.00 ± 2.00–4.00	456
Anterior part of palate^b^	2.88 ± 1.31	3.00 ± 2.00–4.00	444
Inside cheeks^a,b,c^	2.51 ± 1.32	2.00 ± 1.00–4.00	447
Lower lip^b,d^	2.84 ± 1.30	3.00 ± 2.00–4.00	448
Anterior part of tongue^a,d,e^	2.96 ± 1.33	3.00 ± 2.00–4.00	445
Posterior part of tongue^d,e^	2.99 ± 1.37	3.00 ± 2.00–4.00	452
Floor of the mouth^a,b,c,e,f,g^	2.58 ± 1.35	3.00 ± 1.00–4.00	445

Data are presented as median with corresponding interquartile range (IQR) and as a mean with standard deviation (SD)

^a^Wilcoxon signed-rank tests: *p* < 0.05 vs. upper lip

^b^Wilcoxon signed-rank tests: *p* < 0.05 vs. posterior palate

^c^Wilcoxon signed-rank tests: *p* < 0.05 vs. anterior palate

^d^Wilcoxon signed-rank tests: *p* < 0.05 vs. inside cheeks

^e^Wilcoxon signed-rank tests: *p* < 0.05 vs. lower lip

^f^Wilcoxon signed-rank tests: *p* < 0.05 vs. anterior part of the tongue

^g^Wilcoxon signed-rank tests: *p* < 0.05 vs. posterior part of the tongue

### Various dry-mouth patient groups

The European Medical Risk-Related History questionnaire was completed by 517 patients in the total study population. On the basis of their health status, we distinguished seven different groups of patients (see Table 1). All patient groups were included in the statistical comparisons, except for the RTX + High Med group, due to its small number of patients (*N* = 6).

Table 4 shows the different patient characteristics for all six patient groups. Low Med patients were the largest group, and RTX patients were the smallest. High Med patients had the highest mean age, while controls had the lowest.

**Table 4:** Patients were divided into six different patient groups based on their health status

Patient groups (*N* = 517)	Total subjects for each patient group	Age: mean ± SD*	% of woman/men*	Number of medication; median ± IQR *	Total XI-scores: median ± IQR*	UWS: median ± IQR*	pH UWS: median ± IQR	CH-SWS: median ± IQR*	pH CH-SWS: median ± IQR	A-SWS: median ± IQR*	pH A-SWS: median ± IQR*
Controls	136	50.6 ± 17.7	64.2: 35.8	–	27.0 ± 19.0–34.0	0.22 ± 0.07–0.36	6.50 ± 6.10–7.00	0.76 ± 0.44–1.33	7.00 ± 6.70–7.00	1.62 ± 1.01–2.20	4.70 ± 4.40–5.80
Low Med patients	157	60.7 ± 14.8^a^	68.2: 31.8	2 ± 1 – 2^a^	30.0 ± 22.0–37.0^a^	0.17 ± 0.07–0.32	6.50 ± 6.10–7.00	0.72 ± 0.39–1.18	7.00 ± 6.50–7.00	1.17 ± 0.71–1.80^a^	4.70 ± 4.00–6.10
High Med patients	140	65.9 ± 13.1^a,b^	65.7: 34.3	6 ± 4 – 9^a,b^	33.0 ± 24.0–40.0^a,b^	0.11 ± 0.04–0.30^a,b^	6.5− ± 5.80–6.90	0.57 ± 0.27–1.08^a^	7.00–6.10-7.00	0.95 ± 0.44–1.50^a,b^	4.40 ± 4.00–5.00^a,b^
RTX patients	10	58.7 ± 17.9	40.0: 60.0	1 ± 0 – 1^a,b,c^	37.5 ± 31.0–43.8^a,b^	0.13 ± 0.05–0.23	6.10 ± 5.65–6.90	0.45 ± 0.29–0.76	7.00 ± 6.40–7.00	0.52 ± 0.24–0.95^a,b^	4.55 ± 4.15–5.08
SS patients	46	61.7 ± 14.0^a^	84.8: 15.2^a,b,c,d^	2 ± −2^a,b,c,d^	44.0 ± 37.0–49.8^a,b,c^	0.08 ± 0.03–0.16^a,b^	6.50 ± 6.00–6.80	0.30 ± 0.05–0.61^a,b,c^	6.90 ± 6.10–7.00	0.50 ± 0.23–1.15^a,b,c^	4.40 ± 4.00–5.00^a,b^
SS + High Med patients	22	62.1 ± 9.1^a^	95.5: 4.5^a,b,c,d^	7 ± 5 – 9^a,b,d,e^	46.0 ± 37.5–49.5^a,b,c,d^	0.06 ± 0.03–0.16^a,b^	6.50 ± 5.50–7.00	0.28 ± 0.14–0.56^a,b,c^	7.00 ± 6.50–7.00	0.48 ± 0.23–1.35^a,b^	4.40 ± 4.00–4.70^a,b^

Several patient characteristics for the six groups are shown; age, distribution of women and men in each group, the total number of medications used, the total XI-scores, salivary flow rates (in mL/min), and the salivary pH of UWS, CH-SWS, and A-SWS. The age is presented as mean with standard deviation (SD). For the number of medications used, the total XI-scores, salivary flow rates, and salivary pH, the median with corresponding interquartile range (IQR) is shown. The distribution of women and men is given in percentages

*Significant differences between the six patient groups, Kruskal-Wallis test *p* < 0.01

^a^Mann-Whitney *U* test: *p* < 0.05 vs. controls

^b^Mann-Whitney *U* test: *p* < 0.05 vs. Low Med patients

^c^Mann-Whitney *U* test: *p* < 0.05 vs. High Med patients

^d^Mann-Whitney *U* test: *p* < 0.05 vs. RTX patients

^e^Mann-Whitney *U* test: *p* < 0.05 vs. SS patients

There were significantly higher percentages of women in the SS and SS + High Med patient groups than in the other four patient groups (Table 4).

The number of medications used also differed significantly among the six patient groups; High Med and SS + High Med patients used the highest number of medications. All other patient groups used between zero and two medications (Table 4).

Controls had significantly lower total XI-scores than all other groups, indicating that the overall dry-mouth feeling they experienced was less. On the other hand, SS and SS + High Med patients had the highest XI-scores, indicating that their dry-mouth feeling was significantly more severe than that of controls, Low Med patients, and High Med patients (Table 4).

With regard to the salivary flow rates, there was a trend whereby controls and Low Med patients had the highest salivary flow rates for UWS, CH-SWS, and A-SWS, while SS and SS + High Med patients had the lowest (Table 4). The difference among the patient groups with the highest and lowest flow rate was significant for UWS, CH-SWS, and A-SWS. Only the pH of A-SWS differed significantly from that in the various patient groups, being significantly higher in controls and Low Med patients than in SS and SS + High Med patients.

Overall, these results indicate that controls and Low Med patients had the highest salivary flow rates and pH. These groups also experienced less overall dry-mouth feeling as measured with the XI. On the other hand, SS and SS + High Med patients had the lowest salivary flow rates and pH, indicating that their salivary glands produced less saliva and that their salivary pH was lower. Further, these patients had the highest XI-scores, indicating that their overall dry-mouth feeling was more severe.

### Regional Oral Dryness Inventory for the various dry-mouth patient groups

Tables 5 and 6 show the perceived oral dryness in eight different intra-oral regions as determined with RODI for the six patient groups. In these patients groups, all eight intra-oral regions differed significantly (Kruskal Wallis test, *p* < 0.01).

**Table 5:** Perceived oral dryness in four different intra-oral regions of the upper jaw as determined with the Regional Oral Dryness Inventory (RODI) in six different patient groups

Patient groups	Upper lip: mean ± SD (*N*)*	Upper lip: median ± IQR	Anterior palate: mean ± SD (*N*)*	Anterior palate: median ± IQR	Posterior palate: mean ± SD (*N*)*	Posterior palate: median ± IQR	Inside cheeks: mean ± SD (*N*)*	Inside cheeks: median ± IQR
Controls (*N* = 136)	2.40 ± 1.31 (*N* = 113)	2.00 ± 1.00–3.00	2.34 ± 1.25 (*N* = 114)	2.00 ± 1.00–3.00	2.64 ± 1.23 (*N* = 115)	3.00 ± 1.00–4.00	2.05 ± 1.25 (*N* = 113)	1.00 ± 1.00–3.00
Low Med patients (*N* = 157)	2.68 ± 1.21 (*N* = 134)	3.00 ± 2.00–4.00	2.74 ± 1.26 (*N = 131*)^a^	3.00 ± 2.00–4.00	2.81 ± 1.32 (*N* = 135)	3.00 ± 1.00–4.00	2.28 ± 1.30 (*N* = 130)	2.00 ± 1.00–3.00
High Med patients (*N* = 140)	3.08 ± 1.28 (*N = 121*)^a,b^	3.00 ± 2.00–4.00	3.23 ± 1.35 (*N = 119*)^a,b^	4.00 ± 2.00–4.00	3.23 ± 1.32 (*N = 122*)^a,b^	4.00 ± 2.00–4.00	2.78 ± 1.34 (*N = 121*)^a,b^	3.00 ± 2.00–4.00
RTX patients (*N* = 10)	3.00 ± 1.05 (*N* = 10)	3.00 ± 2.00–4.00	3.00 ± 1.00 (*N* = 9)	3.00 ± 2.50–4.00	3.30 ± 1.42 (*N* = 10)	4.00 ± 1.75–4.00	3.30 ± 0.82 (*N = 10*)^a,b^	3.50 ± 2.75–4.00
SS patients (*N* = 46)	3.40 ± 0.98 (*N = 40*)^a,b^	3.00 ± 3.00–4.00	3.33 ± 1.14 (*N = 40*)^a,b^	4.00 ± 3.00–4.00	3.79 ± 0.90 (*N = 42*)^a,b,c^	4.00 ± 3.00–4.00	2.98 ± 1.07 (*N = 40*)^a,b^	3.00 ± 2.00–4.00
SS + High Med patients (*N* = 22)	3.50 ± 1.04 (*N = 18*)^a,b^	4.00 ± 2.75–4.00	3.72 ± 0.75 (*N = 18*)^a,b^	4.00 ± 3.00–4.00	3.84 ± 0.50 (*N = 19*)^a,b^	4.00 ± 4.00–4.00	3.42 ± 1.07 (*N = 19*)^a,b,c^	4.00 ± 3.00–4.00

Data are presented as median with corresponding interquartile range (IQR) and as a mean with standard deviation (SD)

*N* indicates the total number of subjects per intra-oral region

*Significant differences between the six patient groups; Kruskal Wallis test *p* < 0.01

^a^Mann-Whitney *U* test: *p* < 0.05 vs. controls

^b^Mann-Whitney *U* test: *p* < 0.05 vs. Low Med patients

^c^Mann-Whitney *U* test: *p* < 0.05 vs. High Med patients

^d^Mann-Whitney *U* test: *p* < 0.05 vs. RTX patients

^e^Mann-Whitney *U* test: *p* < 0.05 vs. SS patients

**Table 6:** Perceived oral dryness in four different intra-oral regions of the lower jaw as determined with the Regional Oral Dryness Inventory (RODI) in six different patient groups

Patient groups	Lower lip: mean ± SD (N)*	Lower lip: median ± IQR	Anterior tongue: mean ± SD (N)*	Anterior tongue: median ± IQR	Posterior tongue: mean ± SD (N)*	Posterior tongue: median ± IQR	Floor mouth: mean ± SD (N)*	Floor mouth: median ± IQR
Controls (*N* = 136)	2.38 ± 1.27 (*N* = 111)	2.00 ± 1.00–3.00	2.48 ± 1.33 (*N* = 112)	2.50 ± 1.00–4.00	2.57 ± 1.34 (*N* = 113)	3.00 ± 1.00–4.00	2.01 ± 1.20 (*N* = 112)	2.00 ± 1.00–3.00
Low Med patients (*N* = 157)	2.70 ± 1.28 (*N = 135*)^a^	3.00–1.00-4.00	2.83 ± 1.29 (*N = 132*)^a^	3.00 ± 2.00–4.00	2.79 ± 1.34 (*N* = 134)	3.00 ± 1.00–4.00	2.42 ± 1.31 (*N = 132*)^a^	2.00 ± 1.00–4.00
High Med patients (*N* = 140)	3.05 ± 1.27 (*N = 120*)^a,b^	3.00 ± 2.00–4.00	3.25 ± 1.29 (*N = 118*)^a,b^	3.00 ± 2.75–4.00	3.24 ± 1.30 (*N = 120*)^a,b^	3.00 ± 2.00–4.00	2.91 ± 1.35 (*N = 118*)^a,b^	3.00 ± 2.00–4.00
RTX patients (*N* = 10)	3.10 ± 1.10 (*N* = 10)	3.50 ± 2.00–4.00	3.00 ± 1.41 (N = 10)	4.00 ± 1.00–4.00	3.10 ± 1.37 (*N* = 10)	3.50 ± 1.75–4.00	2.70 ± 1.16 (*N* = 10)	3.00 ± 1.75–4.00
SS patients (*N* = 46)	3.40 ± 1.03 (*N = 40*)^a,b^	3.00 ± 3.00–4.00	3.41 ± 1.14 (*N = 41*)^a,b^	4.00 ± 3.00–4.00	3.43 ± 1.21 (*N = 42*)^a,b^	4.00 ± 3.00–4.00	3.07 ± 1.27 (*N = 41*)^a,b^	3.00 ± 2.00–4.00
SS + High Med patients (*N* = 22)	3.53 ± 1.12 (*N = 19*)^a,b^	4.00 ± 3.00–4.00	3.72 ± 1.02 (*N = 18*)^a,b^	4.00 ± 3.00–4.00	4.00 ± 0.67 (*N = 19*)^a,b,c^	4.00 ± 4.00–4.00	3.68 ± 0.95 (*N = 19*)^a,b,c,d^	4.00 ± 3.00–4.00

Data are presented as median with corresponding interquartile range (IQR) and as a mean with standard deviation (SD). *N* indicates the total number of subjects per intra-oral region

*Significant differences between the six patient groups, Kruskal-Wallis test *p* < 0.01

^a^Mann-Whitney *U* test: *p* < 0.05 vs. controls

^b^Mann-Whitney *U* test: *p* < 0.05 vs. Low Med patients

^c^Mann-Whitney *U* test: *p* < 0.05 vs. High Med patients

^d^Mann-Whitney *U* test: *p* < 0.05 vs. RTX patients

^e^Mann-Whitney *U* test: *p* < 0.05 vs. SS patients

The first function of Tables 5 and 6 is to provide an overview of the regions that each of the six patient groups experienced as the most dry and least dry. While the most dry in controls and SS patients was the posterior palate, in Low Med and High Med patients, it was the anterior tongue. The region that was experienced as least dry also differed between groups. In Low Med, High Med, and SS patients, it was the inside cheeks; in controls, it was the floor of the mouth. In RTX and SS + High Med patients, there were no significant differences among the intra-oral regions.

The second function of Tables 5 and 6 is to present the RODI scores for all intra-oral regions for the upper jaw (Table 5) and lower jaw (Table 6). SS and SS + High Med patients had the highest RODI scores for all these regions, while controls and Low Med patients had the lowest. The difference among the patients groups with the highest and lowest RODI scores was significant for all eight intra-oral regions (Mann-Whitney *U* test, *p* < 0.05). This result indicates that SS and SS + High Med patients experienced more severe intra-oral dryness than controls and Low Med patients.

High Med patients experienced significantly more severe intra-oral dryness than controls and Low Med patients, as shown by the higher RODI scores for all eight regions. On the other hand, High Med and SS patients differed only significantly with regard to the RODI scores of the posterior palate, indicating that SS patients experienced more severe dryness of the posterior palate than High Med patients (Table 5).

The RODI scores highlighted significant differences between High Med and SS + High Med patients for several regions. Higher scores showed that SS + High Med patients experienced more severe dryness in the inside cheeks, posterior tongue, and floor of the mouth than High Med patients did.

RTX patients had a significantly higher RODI score than controls and Low Med patients only for the inside cheeks. The RODI scores of RTX and SS + High Med patients differed significantly for the floor of the mouth, RTX patients having lower RODI scores than SS + High Med patients. This means that RTX patients experienced the floor of the mouth as less dry than SS + High Med patients.

As Tables 5 and 6 also show, SS and SS + High Med patients did not differ significantly, indicating that no clear distinction could be made between these two groups on the basis of their RODI scores.

Together, these results provide important insights into perceived intra-oral dryness in the various dry-mouth patient groups, which differed with regard to the regions they experienced as the most and least dry. Their RODI scores also differed significantly for the various intra-oral regions. The lowest RODI scores indicated that controls and Low Med patients experienced less intra-oral dryness and the highest RODI scores that SS and SS + High Med patients experienced more.

### Relationship between the Regional Oral Dryness Inventory and the Xerostomia Inventory in various dry-mouth patient groups

Table 7 presents the Spearman’s correlation between the intra-oral region scores of the RODI and the total XI-scores for the six patient groups.

**Table 7:** Correlations between the perceived oral dryness in eight different intra-oral regions and the total XI-scores for the six different patient groups

Patient groups	Upper lip	Anterior palate	Posterior palate	Inside cheeks	Lower lip	Anterior tongue	Posterior tongue	Floor mouth
XI total for controls	0.57 (0.43–0.69)**	0.66 (0.53–0.77)**	0.66 (0.54–0.75)**	0.54 (0.38–0.68)**	0.51 (0.36–0.64)**	0.61 (0.48–0.71)**	0.61 (0.48–0.70)**	0.49 (0.33–0.63)**
XI total for Low Med patients	0.43 (0.25–0.58)**	0.43 (0.29–0.57)**	0.47 (0.29–0.62)**	0.52 (0.36–0.67)**	0.47 (0.30–0.62)**	0.58 (0.46–0.68)**	0.56 (0.42–0.68)**	0.48 (0.30–0.65)**
XI total for High Med patients	0.56 (0.39–0.70**	0.64 (0.52–0.75)**	0.62 (0.46–0.75)**	0.61 (0.46–0.73)**	0.51 (0.34–0.66)**	0.52 (0.34–0.67)**	0.59 (0.44–0.72)**	0.56 (0.41–0.70)**
XI total for RTX patients	NS	0.69 (0.12–0.98)*	NS	NS	NS	0.70 (0.00–1.00)*	0.78 (0.11–1.00)*	NS
XI total for SS patients	NS	0.48 (0.14–0.76)**	0.66 (0.46–0.82)**	0.48 (0.15–0.74)**	0.34 (−0.01–0.62)*	0.68 (0.47–0.83)**	0.58 (0.30–0.79)**	0.56 (0.24–0.81)**
XI total for SS + High Med patients	0.59 (0.14–0.88)*	NS	NS	0.57 (0.04–0.90)*	0.57 (0.07–0.87)*	NS	NS	0.63 (0.07–0.88)**

*NS* not significant

The Spearman’s rho correlation coefficient is shown with the bias-corrected accelerated (BCa) 95% confidence interval

* indicates that that the correlation is significant at level 0.05

** indicates that that the correlation is significant at level 0.01

The XI-scores of controls, Low Med patients, and High Med patients correlated significantly with all eight intra-oral regions (Spearman’s rank test, *p* < 0.01). The correlation coefficients of these three patient groups ranged between 0.43 and 0.66 and can be viewed as representing fair to moderate correlations.

The XI-scores of RTX patients correlated significantly with only three regions: the anterior palate and the anterior and posterior tongue (Spearman’s rank test *p* < 0.05). These regions had a moderate to very strong correlation with the total XI-scores (correlation coefficients between 0.69 and 0.78).

For SS patients, all regions except for the upper lip correlated significantly with total XI-scores. The correlation coefficients of these regions ranged between 0.34 and 0.68. As for SS + High Med patients, only the following four regions correlated significantly with the total XI-scores: the upper lip, the lower lip, the inside cheeks, and the floor of the mouth. Their correlation coefficients ranged between 0.57 and 0.63, which can be viewed as representing fair to moderate correlation.

Taken together, these results suggest that the correlations between the total XI-scores of controls, Low Med, and High Med patients and all eight intra-oral regions of the RODI can be considered as fair to moderate. On the other hand, RTX, SS, and SS + High Med patients had only a small number of intra-oral regions that correlated significantly with the total XI-scores. However, these correlations were stronger than the correlations of controls, Low Med, and High Med patients.

## Discussion

The results of this study, in which we explored the RODI questionnaire in specific subgroups of dry-mouth patient groups, showed that the regions of perceived intra-oral dryness differed between the groups. Controls and Low Med patients had the lowest RODI scores and experienced less intra-oral dryness than the other groups of patients. On the other hand, SS and SS + High Med patients had the highest RODI scores, meaning that they experienced more intra-oral dryness.

The RODI scores of our total study population revealed that the posterior palate was experienced as the most dry, while the inside cheeks were experienced as the least dry. This result is consistent with the findings of a previous study in which patients also indicated that the posterior palate was the most dry [13].

Several factors make the palate more susceptible to oral dryness than other intra-oral locations: gravity, evaporation, and the paucity of palatal glands [23–25]. For the region that was experienced as the least dry, perceived dryness did not differ significantly between the inside cheeks and the floor of the mouth (Table 3). Both regions include orifices of the major salivary glands [23]. Because of their proximity to the orifices of the salivary glands, the saliva film in these regions is probably more moisturizing than the saliva film on the palate [24, 26–28]. For this reason, all patients experienced the inside cheeks and the floor of the mouth as less dry. This finding is comparable with that in the previous study, which found that patients experienced the floor of the mouth as the least dry [13].

Our results showed that the controls and SS patients experienced the posterior palate as the driest. Notably, they show that SS patients had significantly higher RODI scores (median score 4.00) for the posterior palate than controls did (median score 3.00). This can be explained by the fact that except for the palatal salivary flow rate [29, 30], the UWS flow rate in SS patients is reduced [20, 29–34]. Indeed, the number of patients with xerostomia was higher in SS patients [29, 30, 32]. A plausible explanation is that the subjective feeling of xerostomia is strongly related to the UWS flow. In controls—who had sufficient UWS—the palatal glands contributed little to the dry-mouth feeling [28]. This suggestion is further supported by Wang and co-workers, who did not find a significant correlation between summated XI-scores and minor salivary-gland flow rates [35]. This is consistent with the fact that under healthy conditions, the saliva secreted by the minor salivary glands accounts for less than 10% of whole saliva [36]. Additionally, SS patients have other saliva-related characteristics that induce dry mouth: an altered sialochemical composition, such as higher concentrations of sodium, chloride, and phosphate [20]; a higher protein concentration on the palate [37]; a significantly reduced saliva film on the hard palate; a reduced spinnbarkeit of UWS; and an altered glycosylation of salivary mucins [38]. In conclusion, a drier mouth could be induced in SS patients when altered rheological properties of saliva, reduced mucosal hydration (due to a reduced saliva film), and altered glycosylation combine to cause functional loss of the salivary coating and the lubricating properties of saliva [38].

Low Med and High Med patients experienced the anterior tongue as the most dry. Other studies reported that the thickness of saliva film on the anterior tongue was significantly less in dry-mouth patients—including those with medication-induced hypofunction—than in healthy controls [28, 31, 37, 39]. The saliva-film thickness on the anterior tongue was approximately half of that in controls. In some dry-mouth patients who could not secrete unstimulated saliva, it was even less than half [28]. This finding was confirmed by another study that indicated that oral mucosal wetness varied with the resting salivary flow rate; the lower the flow rate, the thinner the salivary film [27]. Thus, xerostomia emerged when the salivary flow rate was half of its normal value [9, 40, 41].

Reduction of the salivary flow rate and thereby a reduced salivary film thickness on the anterior tongue might therefore explain why Low Med and High Med patients experienced the anterior tongue as the most dry. Besides, the threshold for perceiving dryness is about ≤ 10 μm—the same as that seen in the study of Lee and co-workers [28]. The significantly lower salivary flow rates in High Med patients than in controls (see Table 4) may have induced a very low saliva-film thickness on the anterior tongue below this threshold, thereby causing dryness of the tongue.

Some of the controls in our study had a low salivary flow rate and at times even had hyposalivation of UWS and CH-SWS (see Table 4). Explanations for this may lie in these participants’ age and the possibility that participants had systemic disorders other than Sjögren’s syndrome that were associated with salivary dysfunction. The salivary flow rate in the elderly, even those not using systemic drugs, was significantly lower, especially in non-medicated women in the 45–54 age groups [42]. This finding corresponds with the mean age in our control group (50.6 ± 17.7 years), in which most participants were female (64.2%). Other systemic conditions such as endocrine disorders (diabetes mellitus), neurological disorders (Parkinson’s disease), and metabolic disorders (dehydration) have also been associated with a reduced salivary flow rate [1].

Within our study population, the SS and SS + High Med patients had the lowest salivary flow rates and a reduced pH of A-SWS: proof of hypofunction of the salivary glands. As one would expect, these patient groups also had the highest XI-scores and RODI scores for all intra-oral regions. The severe mouth dryness (both overall dry-mouth experience and intra-oral dryness) they experienced may have been due to the reduced flow rate, but also to altered rheological properties of saliva, and altered glycosylation of mucins.

The RODI questionnaire nonetheless seemed capable of differentiating between dry-mouth patient groups. For example, SS patients could easily be differentiated from controls, Low Med, and High Med patients, as Low Med and High Med patients experienced the anterior tongue as the most dry, while SS patients experienced the posterior palate as the most dry. On the other hand, SS patients had more severe dryness of the posterior palate than controls. These differences in intra-oral dryness can only be diagnosed using the RODI questionnaire and not the XI, as the latter is used only to diagnose the overall dry-mouth experience. For this reason, the RODI questionnaire may be a valuable tool in dry-mouth diagnostics.

It is interesting to note that there were no significant differences between RODI scores in RTX patients. Even when these scores were compared with those of other patient groups, few regions showed intra-oral differences. These results might be related to a lack of statistical power, as the RTX group only comprised 10 subjects. However, RTX patients are not usually difficult to identify, because they can indicate whether they have been treated with radiotherapy of the head and neck region. Most patients will also have been referred to their dentist before and after radiotherapy [43, 44].

With regard to the association between the RODI score and the total XI-scores in various dry-mouth patients, the correlations in the RTX, SS, and SS + High Med patient groups were stronger than the other patient groups. The correlations for these patients were especially strong for the floor of the mouth and for the anterior and posterior tongue (Table 7). These correlations indicate that patients with a very dry mouth overall (higher XI-scores) will also experience more severe oral dryness on the floor of the mouth, and on the anterior and posterior tongue (higher RODI scores for these regions). A previous study that used the Clinical Oral Dryness Score (CODS), a clinical tool to semi-quantitatively assess oral dryness, also found that the CODS items “No saliva pooling in the floor of mouth” and “Tongue fissured” scored higher in the hyposalivation group [18]. This idea was supported by Osailan and co-workers, who reported that the clinical features of oral dryness that are included in the CODS—such as fissured or depapillated tongue, and lack of saliva pooling in the floor of the mouth—are recognized signs of hyposalivation [31]. Other clinical features of their study, such as a mirror sticking to the tongue, a lack of saliva pooling in the floor of the mouth, and a tongue showing loss of papillae, can be associated with a moderate but significant reduction in mucosal wetness [31]. The combination of their results with ours confirms that an important role in dry-mouth perception may be played by two regions: the floor of the mouth and the anterior and posterior tongue. Potentially, the RODI questionnaire would thus play a useful role in early dry-mouth screening, when a patient could be asked specifically about dryness of the floor of the mouth, and of the anterior and posterior tongue. If high RODI scores (score ≥ 3) are obtained for these regions, further dry-mouth diagnostics may be implemented.

A possible limitation of the current study is that the patients included were allocated to the various dry-mouth patient groups on the basis of their self-reported answers to the European Medical Risk-Related History questionnaire [16, 17]. A patient’s health status was thus dependent on his or her reportage. In most cases, there was no confirmation by a physician or a pharmacist either that the patient had Sjögren’s syndrome, or had been irradiated in the head and/or neck region, or about the number of prescription medications that were used. While this information was sometimes confirmed in the referral letter or a medication overview provided by a pharmacist, it was not always available for all patients. The data of this study therefore need to be interpreted with caution. However, the European Medical Risk-Related History questionnaire has a high validity. In previous studies that compared the results of this questionnaire with those of a verbal history taken by a physician experienced in pre-assessment control, sensitivity ranged between 88% and 92%, and specificity was 98–99% [45, 46].

Another possible limitation of the current study is the bias that may have resulted from our collection of saliva at the beginning of a working day, when the unstimulated flow rate changes most rapidly [21]. However, as all patients had been randomly assigned to time slots between 8:00 and 12:00, this potential bias was evenly distributed over the total study population.

## Main conclusions

The present study shows that the RODI questionnaire was able to identify differences between perceived intra-oral dryness in various dry-mouth patient groups. Dry-mouth patients differed with regard to the regions they experienced as the most and least dry. Controls and SS patients experienced the posterior palate as the most dry, and Low Med and High Med patients the anterior tongue. The RODI scores for the various intra-oral regions differed significantly among dry-mouth patients. SS and SS + High Med patients had the highest RODI scores for all intra-oral regions, while controls and Low Med patients had the lowest. These findings suggest that the RODI questionnaire might be a useful additional diagnostic tool for dry-mouth diagnostics, as it may be used to discriminate between potential causes of oral dryness in patients. With the help of this questionnaire, SS patients could be easily differentiated from controls, Low Med, and High Med patients.

The RODI might play an important role in early dry-mouth diagnostics as the floor of the mouth, and the anterior and posterior tongue of the RODI may play important roles in dry-mouth perception.
